# 

**DOI:** 10.1055/s-0035-1570108

**Published:** 2016-01

**Authors:** Carolina Yumi Fujimoto, Rafaela Alkmin da Costa, Tatiana de Assunção Zaccara, Cristiane de Freitas Paganotti, Rossana Pulcineli Vieira Francisco

**Affiliations:** 1 Divisão de Obstetrícia do Hospital das Clínicas da Faculdade de Medicina da Universidade de São Paulo, São Paulo, SP, Brasil; 2 Disciplina de Obstetrícia do Departamento de Obstetrícia e Ginecologia da Faculdade de Medicina da Universidade de São Paulo, São Paulo, SP, Brasil

**Keywords:** frutosamina, hemoglobina A glicosilada, automonitorização da glicemia, diabetes mellitus, gravidez, fructosamine, glycosylated hemoglobin A, blood glucose self-monitoring, diabetes mellitus, pregnancy

## Abstract

**Objetivo** Avaliar se há correlação das dosagens de frutosamina e de hemoglobina glicosilada (HbA1c) com as frequências de desvios de glicemia capilar em gestantes com diabetes mellitus. Métodos: estudo observacional, retrospectivo, de corte transversal, incluindo todas as gestantes com diabetes que iniciaram o pré-natal em hospital terciário de ensino durante o ano de 2014 e que apresentavam pelo menos 20 dias de auto monitoramento glicêmico previamente às dosagens séricas de frutosamina e de HbA1c. Os desvios de glicemia capilar foram considerados “hipoglicemias” quando menores que 70mg/dL ou “hiperglicemias” quando acima do alvo glicêmico terapêutico para o horário. Foram testadas as correlações lineares par a par das dosagens de frutosamina e de HbA1c com as frequências de hipoglicemias e de hiperglicemias capilares pelo teste Tau-b de Kendall. Na sequência, foi avaliada a regressão linear entre as dosagens de HbA1c e de frutosamina e as frequências de hipoglicemias e de hiperglicemias.

**Resultados** Foram incluídas 158 gestantes que contribuíram com 266 amostras para dosagem sérica de frutosamina e HbA1c. As dosagens de frutosamina e de HbA1c apresentaram, respectivamente, coeficientes τ de Kendall de 0,29 ( *p* < 0,001) e 0,5 ( *p* < 0,001) com a frequência de hiperglicemias, e de 0,09 ( *p* = 0,04) e 0,25 ( *p* < 0,001) com a frequência de hipoglicemias capilares. No modelo de regressão linear, as dosagens de frutosamina e de HbA1c apresentaram, respectivamente, coeficientes de determinação R ^2^ = 0,26 ( *p* < 0,001) e R ^2^ = 0,51 ( *p* < 0,001) para a predição de hiperglicemias, e R ^2^ = 0,03 ( *p* = 0,003) e R ^2^ = 0,059 ( *p* < 0,001) para a predição de hipoglicemias.

**Conclusão** As dosagens de frutosamina e de HbA1c apresentam correlação fraca a moderada com as frequências de hiperglicemias e hipoglicemias capilares no auto monitoramento glicêmico e não são capazes de traduzir com precisão os desvios da meta glicêmica no tratamento de gestantes com diabetes.

## Abstract (en)

**Objective** To evaluate the correlation of the levels of fructosamine and of glycated hemoglobin (HbA1c) with the frequency of blood glucose self-monitoring values out of the treatment target range in pregnant women with diabetes mellitus.

**Methods** We performed an observational, retrospective, cross-sectional study, including all pregnant women with diabetes who attended prenatal care visits at a tertiary teaching hospital during the year of 2014 and who presented at least 20 days of blood glucose self-monitoring prior to assessment of serum levels of fructosamine and HbA1c. Capillary blood glucose values out of the treatment target range were considered “hypoglycemia” when lower than 70 mg/dL and “hyperglycemia” when above the glycemic therapeutic target. We evaluated the correlation of the levels of fructosamine and of HbA1c with the frequencies of hyperglycemia and hypoglycemia recorded in the glucometer device by performing Tau-b of Kendall correlation tests. Next, linear regression tests were performed between the levels of HbA1c and of fructosamine and the frequencies of hypoglycemia and hyperglycemia.

**Results** We included 158 pregnant women, from whom 266 blood samples were obtained for assessing fructosamine and HbA1c levels. Measurements of fructosamine and of HbA1c presented, respectively, Kendall's τ coefficient of 0.29 ( *p* < 0.001) and 0.50 ( *p* < 0.001) regarding the frequency of hyperglycemia, and of 0.09 ( *p* = 0.046) and 0.25 ( *p* < 0.001) regarding the frequency of hypoglycemia. In the linear regression model, levels of fructosamine and of HbA1c respectively presented determination coefficients R ^2^ = 0.265 ( *p* < 0.001) and R ^2^ = 0.513 ( *p* < 0.001) for the prediction of hyperglycemia, and R ^2^ = 0.033 ( *p* = 0.003) and R ^2^ = 0.059 ( *p* < 0.001) for the prediction of hypoglycemia.

**Conclusion** Levels of fructosamine and of HbA1c presented a weak to moderate correlation with the frequencies of hyperglycemia and hypoglycemia at blood glucose self-monitoring and were not able to accurately translate the deviations from the glycemic goals in pregnant women with diabetes.

## Introdução

O diabetes *mellitus* (DM) na gravidez está associado a maiores taxas de abortamento, malformações fetais, pré-eclâmpsia, distúrbios na produção de líquido amniótico, desvios do crescimento fetal, sofrimento fetal, tocotrauma, parto cesárea, mortalidade perinatal e complicações neonatais, como hipoglicemia, hiperbilirrubinemia, hipocalcemia e desconforto respiratório do recém-nascido. O risco dessas complicações aumenta de forma diretamente proporcional aos níveis maternos de glicose plasmática. 1 2 3

A melhor forma de se prevenir as complicações perinatais é evitar hiperglicemias durante a gestação. Fora da gestação, o método mais frequentemente utilizado para se avaliar o controle de glicemia das pacientes é a média da glicemia sérica, muitas vezes inferida pela dosagem da hemoglobina glicosilada (HbA1c). Sabe-se, contudo, que um determinado valor de HbA1c pode estar associado a diferentes valores de amplitude da variação glicêmica ao longo de um mesmo dia e entre dias diferentes. 4

Além da HbA1c, diversas outras proteínas passam pelo processo de glicosilação não enzimática. A concentração sérica de algumas dessas proteínas também pode ser usada para estimar o controle glicêmico. O termo “frutosamina” refere-se às cetoaminas formadas neste processo, especialmente representadas pela albumina. A taxa de renovação da albumina sérica é mais rápida que a da hemoglobina (28 × 120 dias), de forma que a frutosamina sérica refletiria um período de tempo menor que a HbA1c 4 e poderia, portanto, ser utilizada como parâmetro auxiliar para o controle glicêmico de portadores de DM em situações nas quais a aplicabilidade da HbA1c fosse limitada, como em hemoglobinopatias, hemólise e anemia, representando a concentração média da glicose plasmática das últimas três semanas. No entanto, assim como a HbA1c, a medida da frutosamina também refletiria uma média glicêmica, podendo não representar tão bem as excursões glicêmicas do período avaliado, desvios de glicemia que podem interferir nos desfechos gestacionais. 5

A literatura é controversa em demonstrar o grau de correlação entre as dosagens de frutosamina e de HbA1c na gestação 6 7 8 9 10 e ainda mais escassa sobre a correlação entre estas proteínas glicosiladas e os desvios de glicemia capilar, 11 tão relevantes durante o tratamento de gestantes com diabetes.

O objetivo do presente estudo é o de avaliar a correlação entre as dosagens de HbA1c e de frutosamina durante a gestação, bem como investigar se os níveis destes produtos de glicosilação apresentam correlação significativa e relação linear com a frequência de desvios glicêmicos durante o tratamento do diabetes durante a gravidez.

## Métodos

Foi realizado estudo observacional, retrospectivo, de corte transversal, envolvendo gestantes portadoras de diabetes gestacional ou pré-gestacional que iniciaram o pré-natal no setor de endocrinopatias na gestação da Clínica Obstétrica do Hospital das Clínicas da Faculdade de Medicina da Universidade de São Paulo (HC-FMUSP) durante o ano de 2014. Foram incluídas todas as gestantes diabéticas, gestacionais ou pré-gestacionais, acompanhadas no serviço neste período e que apresentassem pelo menos 20 dias de auto monitoramento glicêmico previamente à dosagem sérica de frutosamina e de HbA1c. Gestantes que apresentassem mais de uma coleta de sangue precedida por pelo menos 20 dias de registro de automonitoramento glicêmico contribuíram com mais de uma amostra para o estudo. Por tratar-se de análise retrospectiva de dados, dispensou-se a aplicação de termo de consentimento às gestantes.

As gestantes com diagnóstico de diabetes foram acompanhadas de acordo com o protocolo assistencial institucional para o tratamento durante a gestação. De acordo com este protocolo, o controle da glicemia é realizado por meio de dieta, com ou sem insulinoterapia, conforme necessário para que se atinjam as metas glicêmicas preconizadas: 95 mg/dL em jejum, 140 mg/dL 1h após as refeições e 100 mg/dL antes das refeições e na madrugada. 12 13 Para isso, todas as gestantes são orientadas a realizar o automonitoramento glicêmico, aferindo a glicemia capilar de quatro a sete vezes ao dia, a depender do uso de insulina. Este automonitoramento glicêmico é registrado em glicosímetro individual com memória, que é conferido pelo médico nas consultas de pré-natal e registrado em banco de dados informatizado. São identificadas e quantificadas em frequência relativa (porcentagem) as glicemias que estão fora da meta terapêutica, sendo consideradas “hiperglicemias” aquelas que estão acima da meta terapêutica para o horário de aferição e “hipoglicemias” as medidas abaixo de 70 mg/dL. Estes desvios de glicemia são avaliados em cada consulta e norteiam o tratamento das gestantes (como introdução ou ajuste de dose de insulina, internação clínica e indicação de parto). 13

Também conforme o protocolo assistencial da instituição, a cada dois a três meses são realizadas dosagens séricas de frutosamina e de HbA1c para acompanhamento adicional do controle metabólico, 13 as quais são analisadas no laboratório central do HC-FMSUP, respectivamente, pelas técnicas de estudo colorimétrico automatizado (análise quantitativa baseada na comparação da cor produzida por uma reação química com uma cor padrão, através do uso de um espectrofotômetro. De acordo com a intensidade da cor produzida, infere-se a concentração da substância que se quer analisar) e de cromatografia líquida de alta eficiência (método físico-químico de separação que se fundamenta na migração diferencial dos componentes de uma mistura. É usada principalmente para fracionar hemoglobinas e hemoglobina glicada. Pode ser usada para proteínas, peptídeos e aminoácidos), com valores de referência fornecidos pelo laboratório, respectivamente, 205 a 285 umol/L e 4,1–6,0%.

Para a presente análise, os dados foram obtidos por acesso ao sistema informatizado do setor de endocrinopatias na gestação da Clínica Obstétrica do HC-FMUSP e ao sistema de laudos de exames laboratoriais do HC-FMUSP (HCMED).

As dosagens de frutosamina (umol/L) e de HbA1c (%) e as frequências relativas (porcentagens) de hiperglicemias e de hipoglicemias registradas pelo automonitoramento glicêmico nos 20 a 30 dias que precederam as coletas de sangue das gestantes foram apresentadas em forma de média, mediana, valor mínimo e valor máximo.

As distribuições destas variáveis foram testadas para normalidade pelo teste de Kolmogorov-Smirnov. Como não apresentassem distribuição normal, avaliamos as correlações entre pares por meio do teste não paramétrico Tau de Kendall, com coeficientes τ variando de -1 a +1, sendo tanto mais forte a correlação entre as medidas quanto mais próximo de −1 (correlação linear negativa) ou de +1 (correlação linear positiva).

Foram estudadas as correlações entre as dosagens de frutosamina e de HbA1c, entre as dosagens de frutosamina e as frequências de hiperglicemias, entre a dosagens de frutosamina e as frequências de hipoglicemias, entre as dosagens de HbA1c e as frequências de hiperglicemias e entre as dosagens de HbA1c e as frequências de hipoglicemias.

Na sequência, foi realizado teste de regressão linear entre as dosagens de HbA1c e as frequências de hiperglicemias e hipoglicemias e entre as dosagens de frutosamina e as frequências de hiperglicemias e hipoglicemias. Para a análise dos dados foi utilizado Software SPSS v 20.0 e foram considerados significativos valores de *p* < 0,05.

## Resultados

Foram identificadas 337 gestantes com diabetes matriculadas no pré-natal da Clínica Obstétrica do HC-FMUSP no ano de 2014, dentre as quais 158 apresentavam registro de pelo menos 20 dias de automonitoramento glicêmico antes das dosagens séricas de frutosamina e de HbA1c. Destas gestantes foram obtidas 266 amostras sanguíneas ao longo da gestação. Dentre as participantes do estudo, 11 (7%) apresentavam diagnóstico de DM tipo 1, 36 (23%) DM tipo 2, 109 (69%) diabetes gestacional e 2 (1%) *overt diabetes.*

As dosagens de frutosamina e de HbA1c e as frequências de hiperglicemias e de hipoglicemias registradas pelo automonitoramento glicêmico nos 20 a 30 dias que antecederam as amostras séricas estão apresentadas na Tabela 1 .

**Tabela 1:** Resultados das dosagens de frutosamina e de hemoglobina glicosilada (HbA1c) e frequência de excursões glicêmicas pelo automonitoramento de glicemia capilar nas gestantes com diabetes acompanhadas na Clínica Obstétrica do HC-FMUSP no ano de 2014

	Média	Mediana	Mínimo	Máximo
**Frutosamina (umol/L)**	199,1	193,0	117,0	433,0
**Hb A1c (%)**	5,5	5,3	3,8	9,4
**Frequência de Hiperglicemia (%)** a	17,8	19,9	0	71,7
**Frequência de Hipoglicemia (%)** b	3,7	1,4	0	30,6

a glicemia capilar acima do alvo terapêutico, a saber: 95 mg/dL em jejum, 140 mg/dL 1 hora após refeições e 100 mg/dL antes das refeições e na madrugada;

b glicemia capilar menor que 70 mg/dL.

O teste Tau-b de Kendall apresentou correlações lineares positivas e significativas entre todos os pares de interesse, com coeficientes que indicaram correlações fracas a moderadas. Os coeficientes τ de Kendall obtidos foram de τ = 0,19 entre frutosamina e HbA1c ( *p* < 0,001); τ = 0,29 entre frutosamina e frequência de hiperglicemias ( *p* < 0,001); τ = 0,09 entre frutosamina e frequência de hipoglicemias ( *p* = 0,046); τ = 0,50 entre HbA1c e frequência de hiperglicemias ( *p* < 0,001); e τ = 0,25 entre HbA1c e frequência de hipoglicemias ( *p* < 0,001).

**Fig. 1:**
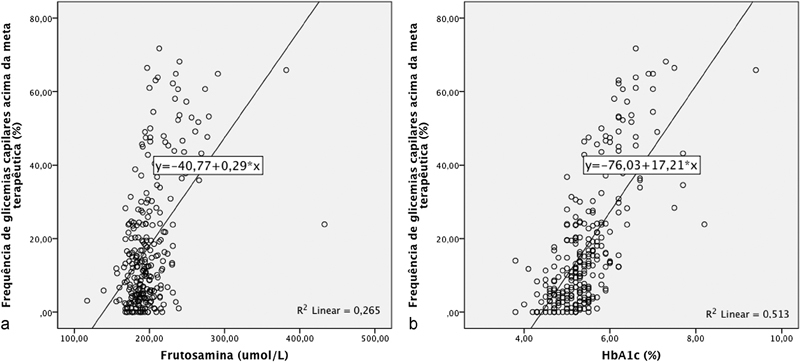
Regressão linear entre as medidas de frutosamina e de hemoglobina glicosilada (HbA1c) com as frequências de glicemias capilares acima da meta terapêutica (“hiperglicemias”) ao automonitoramento glicêmico em gestantes com diabetes acompanhadas na Clínica Obstétrica do HC-FMUSP no ano de 2014 ( *N* = 158).

Em seguida, foi realizado teste de regressão linear entre as dosagens da frutosamina e da HbA1c e as frequências de hiperglicemias, as quais estão apresentadas na Fig. 1 . Para a predição da frequência de hiperglicemias, a medida da frutosamina apresentou coeficiente de determinação R ^2^ = 0,26 ( *p* < 0,001) e a medida da HbA1c apresentou coeficiente de determinação R ^2^ = 0,513 ( *p* < 0,001). Os modelos de predição de frequência de hiperglicemias pelas dosagens de frutosamina e de HbA1c estão apresentados na Tabela 2 . Por ela observa-se que um aumento de 1% na dosagem da HbA1c prediz em 51,3% das vezes um aumento de 17,2% (IC 95% 15,15–19,24, *p* < 0,001) na frequência de hiperglicemias. Analogamente, um aumento de 1 umol/L na dosagem de frutosamina prediz em 26,5% das vezes um aumento de 0,29% (IC 95% 0,24–0,35, *p* < 0,001) na frequência de hiperglicemias ao auto monitoramento glicêmico.

**Tabela 2:** Modelos de regressão linear para predição do percentual de hiperglicemias ao automonitoramento glicêmico pelas dosagens de frutosamina (modelo 1) e de HbA1c (modelo 2) em gestantes com diabetes acompanhadas na Clínica Obstétrica do HC-FMUSP no ano de 2014

Modelo	Variável	B	Erro Padrão	t	Valor de p	IC 95% para B		R ^2^ linear
						Mín	Máx	
1	Constante	−40,77	6,08	−6,71	< 0,001	−52,7	−28,8	0,26
	Frutosamina	0,29	0,03	9,75	< 0,001	0,2	0,3	
2	Constante	−76,03	5,68	−13,40	< 0,001	−87,2	−64,8	0,51
	HbA1c	17,21	1,03	16,68	< 0,001	15,1	19,2	

Abreviações: HbA1c, hemoglobina glicosilada; IC, intervalo de confiança; R ^2^ , coeficiente de determinação do modelo de regressão linear.

Da mesma forma, foi realizado teste de regressão linear entre as dosagens da frutosamina e da HbA1c e as frequências de hipoglicemias registradas pelo auto monitoramento glicêmico. A medida da frutosamina apresentou coeficiente de determinação R ^2^ = 0,033 para predição da frequência de hipoglicemias ( *p* = 0,003) e a medida da HbA1c apresentou coeficiente de determinação R ^2^ = 0,059 para predição da frequência de hipoglicemias ( *p* < 0,001). Os modelos de predição de frequência de hipoglicemias pelas dosagens de frutosamina e de HbA1c estão apresentados na Tabela 3 . Apenas 3,3% da variação na frequência de hipoglicemias pode ser explicada pela variação da dosagem de frutosamina e 5,9% pela variação da HbA1c.

**Tabela 3:** Modelos de regressão linear para predição do percentual de hipoglicemias ao automonitoramento glicêmico pelas dosagens de frutosamina (modelo 1) e de HbA1c (modelo 2) em gestantes com diabetes acompanhadas na Clínica Obstétrica do HC-FMUSP no ano de 2014

Modelo	Variável	B	Erro Padrão	t	Valor de p	IC 95% para B		R ^2^ linear
						Mín	Máx	
1	Constante	−2,10	1,94	−1,08	0,280	−5,93	1,72	0,033
	Frutosamina	0,03	0,01	3,01	0,003	0,01	0,05	
2	Constante	−5,17	2,20	−2,35	0,019	−9,50	−0,84	0,059
	HbA1c	1,62	0,40	4,06	< 0,001	0,83	2,41	

Abreviações: HbA1c, hemoglobina glicosilada; IC, intervalo de confiança; R ^2^ , coeficiente de determinação do modelo de regressão linear.

## Discussão

O diabetes é uma pandemia, que afeta aproximadamente 55% da população mundial. Existem fortes evidências de que a progressão de complicações relacionadas ao diabetes pode ser prevenida ou retardada com bom controle glicêmico. Por este motivo, testes baratos, confiáveis e de fácil aplicabilidade são importantes para o cuidado do paciente diabético. 14

Nossos resultados demonstram correlação linear positiva significativa, porém fraca, entre as dosagens de frutosamina e as frequências de hiperglicemias e de hipoglicemias aferidas pelo automonitoramento de glicemia capilar durante o tratamento de diabetes na gestação. A dosagem de HbA1c, por sua vez, apresentou correlação linear positiva significativa e moderada com a frequência de hiperglicemias e correlação linear positiva significativa e fraca com a frequência de hipoglicemias ao auto monitoramento glicêmico. O modelo de regressão linear apresentou coeficiente de determinação máximo de R ^2^ = 0,513 para predição da frequência de hiperglicemias pela dosagem de HbA1c.

Atualmente, os métodos mais utilizados para monitorar o controle glicêmico são a medida da HbA1c e o auto monitoramento glicêmico com aferição da glicemia capilar. Este último exige a realização de frequentes punções digitais e controle rigoroso de horários e rotinas, já que as medidas devem ser realizadas em conformidade com as refeições. Quanto à HbA1c, apesar de sua comodidade de obtenção, sua dosagem reflete a média glicêmica dos últimos 120 dias, período da meia-vida das hemácias, tendo sua aplicabilidade mais limitada na avaliação de mudanças do controle glicêmico para prazos mais curtos. 4 Além disso, sabe-se que a HbA1c é influenciada pela taxa de renovação celular das hemácias, de forma que valores falsamente elevados podem ser obtidos quando a renovação é baixa, como nos casos de anemia por deficiência de ferro, e valores falsamente baixos podem ser obtidos em situações de renovação aumentada, como em pacientes com hemólise, dislipidemia, ingestão crônica de salicilatos ou a própria gestação. 4 Soma-se à taxa aumentada de renovação celular das hemácias o estado de hemodiluição fisiológica na gravidez e tem-se que neste período são sensivelmente menores os valores de HbA1c obtidos, 15 questionando-se o quanto as variações de HbA1c são capazes de traduzir mudanças do perfil glicêmico.

A frutosamina, por sua vez, apresenta-se como parâmetro auxiliar para a avaliação do controle glicêmico em portadores de diabetes nas situações em que a aplicabilidade da HbA1c fosse limitada. 11 Particularmente, a frutosamina seria capaz de fornecer informações sobre o estado glicêmico para um intervalo de tempo mais curto, característica que seria vantajosa na gestação, quando o prazo para se atingir bom controle glicêmico é menor e intervenções mais dinâmicas são instituídas, promovendo variações mais agudas no perfil glicêmico das gestantes. 6

Apesar de ambos métodos representarem a média glicêmica recente, nossos resultados demonstraram correlação linear positiva significativa, porém fraca, entre as medidas de frutosamina e de HbA1c. A literatura é controversa sobre a força de correlação entre estas medidas durante a gravidez, com alguns autores demonstrando correlação forte 7 8 9 e outros autores demonstrando correlação moderada. 6 10 Uma possível causa para estas divergências é que variações de HbA1c e de frutosamina não representam intervalos de tempo semelhantes. A HbA1c reflete em 50% a glicemia dos últimos 30 dias, em 25% a glicemia dos últimos 30–60 dias e em 25% dos últimos 60–120 dias. Logo, ao se comparar sua dosagem com a da frutosamina, que reflete um intervalo mais recente, de duas a três semanas, pode haver discrepância nos valores apresentados, especialmente em situações de mudanças agudas no perfil glicêmico, a que estão particularmente sujeitas as gestantes diabéticas. Alguns trabalhos 10 16 avaliaram a variação destes marcadores após início de terapia medicamentosa e verificou-se que enquanto a glicemia pré-prandial diminuiu em 72%, a frutosamina teve redução de apenas 58% e a HbA1c somente de 39%, evidenciando diferentes padrões de respostas dos marcadores às variações glicêmicas na gestação. Sabe-se, ainda, que uma ampla e aguda flutuação glicêmica poderia aumentar a glicosilação da albumina, enquanto poderia diminuir a sobrevida da hemácia, de forma que uma variação glicêmica acentuada poderia diminuir HbA1c e aumentar a frutosamina, 17 fazendo-as variar de formas distintas na gravidez.

Para que se considere o controle glicêmico adequado na gestação, visando reduzir as complicações perinatais, as metas terapêuticas atualmente preconizadas pautam-se nos valores de glicemia capilar aferidos pelo auto monitoramento glicêmico, 12 não existindo ainda alvos bem estabelecidos para a média glicêmica, para a dosagem de frutosamina ou para a dosagem de HbA1c durante a gravidez, especialmente para os casos de diabetes gestacional. Uma vez que são medidas mais simples e de maior praticidade de obtenção, a constatação de forte correlação entre medidas de HbA1c ou de frutosamina com as frequências de desvios da glicemia capilar poderia facilitar o seguimento de gestantes com diabetes, sendo estes desvios glicêmicos inferidos a partir do valor da HbA1c ou da frutosamina.

Em nosso estudo, no entanto, a força de correlação dos valores de frutosamina e de HbA1c com a frequência de medidas acima da meta terapêutica (“hiperglicemias”), foi, respectivamente, fraca e moderada. A análise de regressão linear entre estes fatores também demonstrou capacidades de predição fraca para frutosamina e moderada para HbA1c, evidenciando limitação dos métodos como alternativas ao automonitoramento glicêmico.

Em estudo semelhante ao nosso, 11 investigando a correlação entre a dosagem de frutosamina e parâmetros de controle glicêmico, dentre os quais desvios-padrões das glicemias e índices de glicemia acima e abaixo da faixa-alvo, também aferidos pela glicosimetria capilar, não se observou correlação significativa entre a dosagem de frutosamina e a média glicêmica, mas descreveu-se correlação significativa, positiva e fraca entre a frutosamina e o desvio-padrão das glicemias (r = 0,28, *p* = 0,021). No entanto, para os autores a faixa-alvo terapêutica do controle glicêmico foi de 60 a 126 mg/dL independentemente do horário de aferição, distintamente do nosso trabalho, em que os alvos terapêuticos foram estipulados de acordo com o horário de aferição da glicemia capilar, conforme preconizado nos protocolos de assistência a gestantes com diabetes. 13 Desta forma, valores de glicemia capilar que possam ter sido considerados normais para a faixa-alvo terapêutica dos autores poderiam ter sido classificados, em nossa análise, como “hiperglicemia,” pois exigiriam medidas de intervenção para controle glicêmico.

Também é importante ressaltar que tanto a frutosamina como a HbA1c apresentaram fraca correlação com a frequência de hipoglicemias, parâmetro relevante no seguimento das gestantes, especialmente daquelas que fazem uso de insulina e que têm estes eventos relevados para o ajuste da dose do medicamento. Outros autores 11 não evidenciaram correlação significativa entre a dosagem de frutosamina e a frequência de episódios de hipoglicemia.

Em síntese, diante da reconhecida importância de se atingir bom controle glicêmico em gestantes com diabetes, e da limitação por nós constatada em se inferir a frequência de excursões glicêmicas (hiperglicemias e hipoglicemias) pela dosagem da frutosamina ou da HbA1c, reforça-se a importância do automonitoramento glicêmico no tratamento do diabetes na gravidez e a limitação de se utilizar a dosagem da HbA1c ou da frutosamina para o manejo do tratamento do diabetes nestas gestantes.

As dosagens de frutosamina e de HbA1c apresentam correlação fraca a moderada com as frequências de hiperglicemias e hipoglicemias capilares no automonitoramento glicêmico e não são capazes de traduzir com precisão os desvios da meta glicêmica em gestantes com diabetes. Como estes desvios causam impacto nos desfechos gestacionais, o automonitoramento glicêmico não pode ser prescindido pela dosagem da frutosamina ou da HbA1c.
